# Antimicrobial resistance and toxin profiles of enterotoxigenic *Escherichia coli* in children under five years old with diarrhoea in Ouagadougou, Burkina Faso: a sentinel surveillance study

**DOI:** 10.1186/s12879-026-13722-8

**Published:** 2026-06-03

**Authors:** Alimatou Héma, Issa Tondé, Roger Kaboré, Moussa Sangaré, Mamadou Tamboura, Evariste Bako, Touwendsida Serge Bagre, Marguerite Edith Malatala Nikiema, Aminata Ouattara, M. Jahangir Hossain, Subhra Chakraborty, Mahamoudou Sanou

**Affiliations:** 1 Université Joseph KI-ZERBO (UJKZ), Ouagadougou, Burkina Faso; 2 International Center of Excellence in Research in Mali, University of Sciences, Techniques, and Technologies of Bamako, Bamako, Mali; 3 Department of Biochemistry and Microbiology, Centre Universitaire de Tenkodogo, Tenkodogo, Burkina Faso; 4 Université Joseph Ki-Zerbo, Laboratoire de Biologie Moléculaire, d’Epidémiologie et de Surveillance des Bactéries et virus Transmissibles par les Aliments (LaBESTA)/CRSBAN, Centre Universitaire de Ziniaré, Ziniaré, Burkina Faso; 5 Laboratoire de Virologie et Biotechnologies Végétales, Institut de l’Environnement et de Recherches Agricoles (INERA), Ouagadougou, Burkina Faso; 6 Department of International Health, Johns Hopkins Bloomberg School of Public Health, Baltimore, MD 21205 USA; 7 Medical Research Council Unit the Gambia at the London School of Hygiene and Tropical Medicine, Banjul, The Gambia

**Keywords:** Enterotoxigenic *Escherichia coli* (ETEC), Antimicrobial resistance, Multidrug resistance, Paediatric diarrhoea, ESBL, Burkina Faso, West Africa, Antimicrobial stewardship

## Abstract

**Background:**

Enterotoxigenic *Escherichia coli* (ETEC) remains a major cause of paediatric diarrhoeal disease in sub-Saharan Africa. Increasing antimicrobial resistance (AMR) threatens the effectiveness of empirical treatment, yet data on ETEC-specific resistance patterns and toxin profiles in Burkina Faso are scarce. This study assessed the prevalence, toxin gene distribution, and antimicrobial susceptibility of ETEC among children with acute diarrhoea in Ouagadougou, Burkina Faso.

**Methods:**

A hospital-based cross-sectional sentinel surveillance study was conducted at the “Centre Hospitalier Universitaire Pédiatrique Charles De Gaulle (CHUP-CDG)”, Ouagadougou, from May 2023 to April 2024. Stool samples were collected from 383 children under five years of age presenting with acute diarrhoea. ETEC was identified by culture followed by multiplex PCR targeting *eltB* (LT), *estA1* (STp), and *estA2* (STh) genes. Antimicrobial susceptibility testing was performed using the Kirby-Bauer disk diffusion method and interpreted according to EUCAST 2023 criteria. Multidrug resistance (MDR) was defined as non-susceptibility to at least one agent in three or more antimicrobial classes.

**Results:**

ETEC was detected in 16 of 383 children, yielding a prevalence of 4.2% (95% CI: 2.4–6.7). The highest proportion of cases occurred in children aged 12–23 months (43.8% (7/16)). LT-producing strains predominated (50.0% (8/16)), followed by STp-producing strains (43.8%), with one LT + STh isolate (6.3% (1/16)). Antimicrobial resistance was high to commonly used oral agents, including trimethoprim (93.3% (14/15)), sulfamethoxazole (73.3% (11/15)), and ampicillin (66.7% (10/15)). Resistance to third-generation cephalosporins was substantial (ceftazidime 57.1% (8/14), cefotaxime 53.3% (8/15)), and fluoroquinolone resistance reached 40.0% (6/15) for ciprofloxacin. Eleven of 15 tested isolates (73.3% (7/15)) were classified as multidrug-resistant. All isolates remained susceptible to carbapenems and cefoxitin. While 80.0% (12/15) of isolates exhibited a phenotypic profile suggestive of extended-spectrum beta-lactamase (ESBL) production, only one isolate 6.7% (1/15) was phenotypically confirmed as an extended-spectrum beta-lactamase (ESBL) producer by double-disk synergy testing.

**Conclusion:**

The high prevalence of in vitro antimicrobial resistance among ETEC isolates suggests that the effectiveness of commonly used oral antibiotics as empirical treatment options in this setting may be compromised. The 73% MDR prevalence and ESBL production necessitate urgent antimicrobial stewardship implementation, enhanced surveillance systems, and accelerated ETEC vaccine development for this vulnerable population.

**Supplementary Information:**

The online version contains supplementary material available at 10.1186/s12879-026-13722-8.

## Introduction

Diarrhoeal diseases remain a major cause of morbidity and mortality among children under five years of age, particularly in low- and middle-income countries [1, 2]. Despite substantial global progress in reducing diarrhoeal mortality over the past two decades, Sub-Saharan Africa (SSA) continues to bear a disproportionate burden, largely driven by limited access to safe water, sanitation, and timely healthcare [1]. In Burkina Faso, diarrhoeal diseases remain among the leading causes of morbidity and healthcare utilization among children under five years of age. In this context, enteric bacterial pathogens remain important contributors to severe and recurrent diarrhoeal episodes in young children.

Enterotoxigenic *Escherichia coli* (ETEC) is one of the leading bacterial causes of acute watery diarrhoea in infants and young children living in endemic settings, as well as a frequent cause of traveler’s diarrhoea [3]. Globally, ETEC is responsible for an estimated 220 million cases of diarrhoea annually among children under five [4]. In sub-Saharan Africa, ETEC, a key diarrheagenic *E. coli* pathotype, accounts for approximately 10–30% of paediatric diarrhoeal episodes requiring hospitalization, as evidenced by multi-site studies and meta-analyses [5, 6].

ETEC pathogenesis relies on specific colonization factors and the secretion of heat-labile (LT) and/or heat-stable (ST) enterotoxins, which disturb electrolyte balance and fluid absorption in the intestinal mucosa [7]. Strains may produce LT alone, ST alone, or both toxins, and the distribution of toxin profiles varies geographically [8]. This diversity has important implications for disease severity, transmission dynamics, and the effectiveness of ETEC vaccine candidates currently under development.

Recent evidence suggests that the impact of ETEC infection may extend beyond acute diarrhoeal episodes. A systematic review synthesizing data from prospective studies in children under five reported small but consistent associations between ETEC infection and impaired linear growth, highlighting the potential contribution of ETEC to childhood stunting in endemic settings [9]. While evidence for effects on neurocognitive development remains limited and inconsistent, the observed growth detriments reinforce the importance of preventing and effectively managing ETEC infections during early childhood, when growth velocity is highest and vulnerability is greatest [9].

In many resource-limited settings, including Burkina Faso, routine diagnosis of diarrhoeal pathogens depends on culture-based methods, which detect *E. coli* but fail to differentiate pathogenic strains such as ETEC from commensals without molecular confirmation [10, 11]. As a result, the true burden of ETEC infection is likely underestimated, and data on circulating toxin profiles remain sparse. Recent molecular studies using molecular diagnostics have demonstrated that ETEC contributes substantially to paediatric diarrhoea in West Africa [6, 12], yet comprehensive surveillance data remain limited, even in urban referral hospitals.

At the same time, antimicrobial resistance (AMR) among enteric pathogens has emerged as a critical public health threat [13]. Although oral rehydration therapy remains the cornerstone of diarrhoeal disease management [14–16], antibiotics are frequently prescribed in severe cases or when bacterial infection is suspected [17]. In Burkina Faso and neighbouring countries, diarrhoeal management frequently involves aminopenicillins, trimethoprim-sulfamethoxazole, fluoroquinolones, and third-generation cephalosporins [18]. Increasing resistance to these drugs threatens their clinical effectiveness and may contribute to prolonged illness, treatment failure, and increased healthcare utilization [13].

Regional and global surveillance systems, including the WHO Global Antimicrobial Resistance and Use Surveillance System (GLASS), have reported high resistance rates among *Enterobacterales* to first-line antibiotics in Burkina Faso [19–21]. However, these reports largely aggregate data across species and clinical syndromes, providing limited insight into resistance patterns specific to diarrheagenic *E. coli* pathotypes, such as ETEC.

Characterizing current antimicrobial susceptibility patterns of ETEC is particularly important in paediatric populations, where treatment options are already constrained by age-related safety considerations. The emergence of multidrug-resistant (MDR) and extended-spectrum beta-lactamase (ESBL)-producing strains further complicates empirical management and underscores the need for pathogen-specific resistance data to inform treatment guidelines and stewardship interventions [22].

In Burkina Faso, although WHO guidelines recommend primarily supportive management of childhood diarrhoea, antibiotics are often prescribed empirically in the absence of pathogen-specific surveillance data, potentially contributing to inappropriate antimicrobial use and resistance development.

In Burkina Faso, WHO standards for the management of childhood diarrhoea emphasize supportive care, with antibiotics reserved for selected clinical indications [18]. In the absence of routine ETEC surveillance, clinicians often rely on empirical prescribing practices that may be misaligned with local epidemiology, potentially accelerating the development and spread of antimicrobial resistance.

Against this background, updated data on ETEC prevalence, toxin gene distribution, and antimicrobial resistance are urgently needed. This study aimed to determine the prevalence of ETEC among children under five years of age presenting with acute diarrhoea at a tertiary paediatric hospital in Ouagadougou, Burkina Faso; to characterize circulating LT and ST toxin profiles; and to assess antimicrobial susceptibility patterns using standardized EUCAST methodology. By providing contemporary, pathogen-specific surveillance data, this study aims to inform clinical practice, antimicrobial stewardship efforts, and public health strategies, including future ETEC vaccine deployment, in high-burden settings.

## Methods

### Study design and setting

A hospital-based cross-sectional sentinel surveillance study was conducted at the “Centre Hospitalier Universitaire Pédiatrique Charles De Gaulle (CHUP-CDG)”, a tertiary referral center located in Ouagadougou, Burkina Faso. CHUP-CDG provides specialized paediatric care and serves as a major referral hospital for the capital city and surrounding urban health facilities. The study was carried out over a 12-month period, from 1 May 2023 to 30 April 2024.

This study was embedded within routine laboratory diagnostic activities at CHUP-CDG. Stool samples were collected from children presenting with acute diarrhoea whose attending clinicians requested stool culture as part of routine clinical management.

### Study population and eligibility criteria

Study population was constituted by children younger than five years of age presenting with acute diarrhoea for whom stool culture was requested by the attending clinician as part of standard clinical care. Acute diarrhoea was defined as the passage of three or more loose or watery stools within a 24-hour period, with a duration of less than 14 days.

Children were included if a stool sample was submitted to the bacteriology laboratory during the study period and informed consent was obtained from a parent or legal guardian. Samples were excluded if stool volume was insufficient for microbiological analysis or if laboratory contamination or processing failure occurred.

### Sample size determination

The sample size was calculated using the single population proportion formula:

$$n = {Z^2 \times p(1-p)}/{d^2}$$

where Z = 1.96 for a 95% confidence level, *p* = 0.12 based on a previously reported ETEC prevalence in Ouagadougou based on Bonkoungou et al., 2013 [23], and d = 0.05 as the margin of error. The minimum required sample size was 162 participants. To improve the precision of prevalence and antimicrobial resistance estimates and to account for potential specimen rejection or technical failure, enrolment was extended to 383 children during the study period.

### Case definition of enterotoxigenic *Escherichia coli* (ETEC)

An ETEC case was defined as a diarrhoeal stool sample from which *Escherichia coli* was isolated by culture and subsequently confirmed to harbour at least one ETEC-specific enterotoxin gene *eltB* (LT), *estA1* (STp), or *estA2* (STh) by multiplex polymerase chain reaction (PCR).

### Stool culture and bacterial identification

Fresh stool specimens were inoculated onto MacConkey agar (Liofilchem, Roseto degli Abruzzi, Italy) and incubated aerobically at 37 °C for 18–24 h. Lactose-fermenting colonies with morphology suggestive of *Escherichia coli* were subcultured for purity and identified using standard biochemical tests, including Gram staining, catalase, oxidase, indole, citrate utilization, and urease activity. Gram staining reagents were obtained from Scharlau (Scharlab S.L., Sentmenat, Spain). The catalase test was performed using hydrogen peroxide (Laboratoires GILBERT, Hérouville-Saint-Clair, France), and the oxidase test used reagent strips from Liofilchem (Italy). Further biochemical characterization, including indole production, citrate utilization, and urease activity, was assessed using SIM medium, Simmons citrate agar, and urea agar media (Liofilchem, Italy), respectively, following the manufacturers’ instructions for each test. Confirmed *E. coli* isolates were conserved in brain-heart infusion broth supplemented with 25% glycerol and stored at -20 °C until molecular analysis and antimicrobial susceptibility testing were performed.

### DNA extraction and molecular detection of enterotoxigenic *Escherichia coli*

Genomic DNA was extracted from overnight bacterial cultures (18–24 h) using a thermal lysis (boiling) method. Briefly, an isolated bacterial colony was suspended in 100 µL of sterile distilled water in a microcentrifuge tube, incubated at 100 °C for 10 min to induce bacterial lysis, and centrifuged at 14,000 rpm for 2 to 3 min to remove cellular debris. Approximately 90 µL of the supernatant containing the extracted DNA was recovered and used as the PCR template.

Multiplex PCR was performed to detect ETEC enterotoxin genes encoding heat-labile toxin (*elt*), porcine heat-stable toxin (*estA1*), and human heat-stable toxin (*estA2*) according to previously described protocols [11, 24].

Amplification reactions were carried out in a total volume of 20 µL. The reaction mixture consisted of 1 µL of template DNA, 1 µL of each specific primer (Genecust, France), 2.5 µL of Master Mix (Solis Biodyne, Estonia), and 10.5 µL of PCR water (Solis Biodyne, Estonia).

Cycling conditions were performed in a thermal cycler (Applied Biosystems™ SampliAmp™, Thermo Fisher Scientific, Waltham, MA, USA) and consisted of an initial denaturation at 95 °C for 2 min, followed by 30 cycles of denaturation at 95 °C for 15 s, annealing at 52 °C for 30 s, and extension at 72 °C for 10 s. A final extension step was performed at 72 °C for 2 min, followed by a final hold at 10 °C.

Ten microliters (10 µL) of the PCR products were resolved by electrophoresis on a 1.5% (w/v) agarose gel in 1X TBE buffer diluted from a 10X concentrate (Sigma-Aldrich, St. Louis, MO, USA) at 100 V for approximately one hour. The amplicons were visualized and photographed under ultraviolet illumination after staining with RedSafe (Prolabo, Paris, France). Primer sequences and expected amplicon sizes are presented in Table 1.

**Table 1:** Primers used to detect ETEC enterotoxins

Virulence factor	Target gene	Primer name	Primer sequences (5′ to 3′)	Amplicon size (bp)
**LT**	*eltB*	LTF	ACG GCG TTA CTA TCC TCT C	274
		LTR	TGG TCT CGG TCA GAT ATG TG	
**STp**	*estA1*	STpF1	TCT TTC CCC TCT TTT AGT CAG	166
		STpR2	ACA GGC AGG ATT ACA ACA AAG	
**STh**	*estA2*	SThnyF	TTCACCTTTCCCTCAGGATG	120
		SThnyR	CTATTATTCATGCTTTCAGGACCA	

### Antimicrobial susceptibility testing

Antimicrobial susceptibility testing (AST) was performed on all PCR-confirmed enterotoxigenic *Escherichia coli* (ETEC) isolates using the Kirby-Bauer disk diffusion method on Mueller-Hinton agar (Liofilchem^®^, Roseto degli Abruzzi, Italy), in accordance with the Comité de l’Antibiogramme de la Société Française de Microbiologie / European Committee on Antimicrobial Susceptibility Testing (CASFM/EUCAST) 2023 guidelines [25]. Bacterial suspensions were standardized to a 0.5 McFarland turbidity prior to inoculation.

After incubation, inhibition zone diameters were measured and interpreted. Adhering to the updated EUCAST definitions, clinical breakpoints were used to categorize isolates as Susceptible (S), standard dosing regimen (S); Susceptible, increased exposure (I); or Resistant (R). Furthermore, reading and interpretation accounted for the Area of Technical Uncertainty (ATU) where relevant, to mitigate the risk of categorical misclassification for borderline results.

A total of 27 antibiotics were tested, including monobactam: aztreonam (30 µg); penicillins: ampicillin (20 µg), ticarcillin (75 µg); β-lactam/β-lactamase inhibitor combinations: amoxicillin–clavulanic acid (30 µg), ampicillin–sulbactam (20 µg), piperacillin–tazobactam (36 µg), ticarcillin–clavulanic acid (85 µg); cephalosporins: cefuroxime (30 µg), cefoxitin (30 µg); cefotaxime (30 µg), ceftazidime (30 µg), ceftriaxone (30 µg); cefepime (30 µg); aminoglycosides: gentamicin (10 µg), amikacin (30 µg), kanamycin (30 µg), tobramycin (10 µg); carbapenems: imipenem (10 µg), meropenem (10 µg), ertapenem (10 µg); fluoroquinolones: ciprofloxacin (5 µg), levofloxacin (5 µg); macrolide: azithromycin (15 µg); folate pathway inhibitor: trimethoprim (5 µg); sulfonamide: sulfamethoxazole (25 µg); glycylcycline: tigecycline (15 µg); and amphenicol: chloramphenicol (30 µg).

*Escherichia coli* ATCC 25,922 was used as the quality control strain for all antimicrobial susceptibility testing procedures.

Isolates showing resistance to third-generation cephalosporins with preserved susceptibility to cefoxitin were considered phenotypically suggestive of ESBL production.

Confirmatory phenotypic testing using the double-disk synergy test was performed to declare confirmed ESBL strains.

Multidrug resistance (MDR) was defined according to the international standardized definition by Magiorakos et al. (2012) as non-susceptibility (resistant or intermediate) to at least one agent in three or more antimicrobial categories [26]. For the purposes of epidemiological surveillance and MDR calculation, isolates in the “Susceptible, increased exposure” (I) category were retained in the non-susceptible fraction per historical criteria, though their potential clinical utility under optimized dosing is acknowledged.

### Detection of ESBL production and definition of multidrug resistance

Isolates showing resistance to third-generation cephalosporins with preserved susceptibility to cefoxitin were considered phenotypically suggestive of extended-spectrum beta-lactamase (ESBL) production. Confirmatory testing was performed using the double-disk synergy method.

### Ethical considerations

This study was conducted in accordance with the principles of the Declaration of Helsinki. Ethical approval was obtained from the Health Research Ethics Committee (HREC) of Burkina Faso prior to the start of the study (Approval No. 2023-02-037; dated 24 February 2023). Written informed consent was obtained from the parents or legal guardians of all participating children before stool sample collection. All participant data were anonymized to ensure confidentiality.

### Data management and statistical analysis

Descriptive statistics were used to summarize sociodemographic variables. Data analysis was performed using the AMR package in R (version 4.3.1; R Foundation for Statistical Computing, Vienna, Austria). Categorical variables were summarized as frequencies and proportions with 95% confidence intervals. Continuous variables were assessed for normality and summarized using means with standard deviations or medians with interquartile ranges, as appropriate. Antimicrobial resistance analyses were conducted using the AMR package in R [27], applying EUCAST 2023 breakpoints. To identify significant differences in ETEC prevalence, toxin distribution, or resistance patterns across demographic groups (sex and age), comparative analyses were performed using Chi-square or Fisher’s exact tests, as appropriate. A p-value < 0.05 was considered statistically significant. Resistance profiles were visualized using ggplot2 to create bar charts and heatmaps showing resistance patterns across antibiotic classes.

## Results

### Study population characteristics

Between May 1, 2023, and April 30, 2024, 383 children under five years of age presenting with acute diarrhoea were enrolled. Of these, 61.3% (235/383) were male and 38.7% (148/383) were female. The median age was 15 months (IQR = 9–24). Infants aged 0–11 months constituted the largest age group (36.8%), followed by children aged 12–23 months (30.8%). Demographic characteristics of the study population are summarized in Table 2.

**Table 2:** Demographic characteristics of study participants (*N* = 383)

Characteristic	*n* (%)	95% CI
Sex		
Male	235 (61.4%)	56.3–66.3
Female	148 (38.6%)	33.7–43.7
Median (IQR) (months)	15 (9–24)	
Age groups (months)		
0–11 months	141 (36.8%)	32–41.9
12–23 months	118 (30.8%)	26.2–35.7
24–35 months	50 (13.1%)	9.8–16.8
36–47 months	34 (8.9%)	6.2–12.2
48–59 months	40 (10.4%)	7.6–13.9

Note. SD: standard deviation; IQR: interquartile range; CI: confidence interval 95% CI calculated using exact binomial method (Clopper-Pearson) for proportions and t-distribution for means

### Prevalence of enterotoxigenic *Escherichia coli*

Enterotoxigenic *E. coli* was identified in 16 of the 383 stool samples analyzed, corresponding to a prevalence of 4.2% (95% CI: 2.4–6.7%). ETEC detection varied by age group, with the highest proportion observed among children aged 12–23 months (43.8% (7/16)). Nine ETEC-positive cases (56.3% (9/16)) occurred in males and seven (43.7% (7/16)) in females.

### Distribution of ETEC toxin profiles

Among the 16 ETEC PCR confirmed, strains producing heat-labile toxin (LT) alone were most frequent (8/16; 50.0%). Heat-stable toxin producing strains carrying the *estA1* (STp) gene accounted for 7 isolates (43.8% (7/16)). One isolate (6.3% (1/16) harbored both *eltB* (LT) and *estA2* (STh) genes. The distribution of toxin profiles by age group and sex is presented in Table 3.

**Table 3:** Distribution of ETEC cases by age group, sex, and toxin profiles (*n* = 16)

Age group (months)	Total ETEC, *n* (%)	Male, *n* (%)	Female, *n* (%)	LT-ETEC, *n* (%)	LT + STh-ETEC, *n* (%)	STp-ETEC, *n* (%)
**0–11**	3 (18.8)	2 (12.5)	1 (6.3)	1 (6.3)	1 (6.3)	1 (6.3)
**12–23**	7 (43.8)	4 (25.0)	3 (18.8)	4 (25.0)	0 (0.0)	3 (18.8)
**24–35**	2 (12.5)	1 (6.3)	1 (6.3)	2 (12.5)	0 (0.0)	0 (0.0)
**36–47**	3 (18.8)	1 (6.3)	2 (12.5)	1 (6.3)	0 (0.0)	2 (12.5)
**48–59**	1 (6.3)	1 (6.3)	0 (0.0)	0 (0.0)	0 (0.0)	1 (6.3)
**Total**	16 (100.0)	9(56.3)	7(43.7)	8(50.0)	1 (6.3)	7 (43.8)

Note. LT: heat-labile enterotoxin; STh: human heat-stable enterotoxin; STp: porcine heat-stable enterotoxin. Percentages (%) are calculated based on the total number of ETEC isolates (*n* = 16)

The overall toxin gene distribution is illustrated in Supplementary materials.

### Antimicrobial susceptibility patterns

Antimicrobial susceptibility testing (AST) was performed on 15 PCR-confirmed isolates. Resistance to commonly used oral antibiotics was widespread (Table 4). High resistance rates were observed for trimethoprim (93.3% (14/15)), sulfamethoxazole (73.3% (11/15)), ticarcillin (86.7% (13/15)), and ampicillin (66.7% (10/15)).

All isolates were susceptible to imipenem, meropenem, and cefoxitin. Gentamicin susceptibility was 86.7%.

Azithromycin resistance reached 40.0% (6/15), with 60.0% (9/15) susceptible. This high rate threatens its utility as an alternative for multidrug-resistant paediatric ETEC infections in Ouagadougou. Detailed susceptibility results by antimicrobial class are presented in Table 4 and Supplementary materials.

**Table 4:** Antimicrobial susceptibility profiles of 15 ETEC isolates (EUCAST 2023)

Antibiotic class	Antibiotic	Susceptible *n*(%)	Intermediate *n*(%)	Resistant *n*(%)
**Aminoglycosides**	Amikacin (AMK)	11 (73.3)	3 (20.0)	1 (6.7)
	Gentamicin (GEN)	13 (86.7)	0 (0.0)	2 (13.3)
	Kanamycin (KAN)	8 (53.3)	6 (40.0)	1 (6.7)
	Tobramycin (TOB)	8 (53.3)	4 (26.7)	3 (20)
**Amphenicols**	Chloramphenicol (C)	13 (86.7)	1 (6.7)	1 (6.7)
**Carbapenems**	Ertapenem (ETP)	15 (100)	0 (0.0)	0 (0.0)
	Imipenem (IPM)	15 (100)	0 (0.0)	0 (0.0)
	Meropenem (MEM)	15 (100)	0 (0.0)	1 (6.7)
**Cephalosporins**				
**2nd Generation**	Cefoxitin (FOX)	15 (100.0)	0 (0.0)	0 (0.0)
	Cefuroxime (CXM)	8 (53.3)	0 (0.0)	7 (46.7)
**3rd Generation**	Cefotaxime (CTX)	6 (40.0)	1 (6.7)	8 (53.3)
	Ceftazidime (CAZ)	2 (14.3)	4 (28.6)	8 (57.1)
	Ceftriaxone (CRO)	9 (60.0)	0 (0.0)	6 (40.0)
**4th Generation**	Cefepime (FEP)	8 (53.3)	0 (0.0)	7 (46.7)
**Sulfonamides**	Sulfamethoxazole (SMX)	4 (26.7)	0 (0.0)	11 (73.3)
**Folate Pathway Inhibitors**	Trimethoprim (TMP)	1 (6.7)	0 (0.0)	14 (93.3)
**Macrolides**	Azithromycin (AZM)	9 (60.0)	0 (0.0)	6 (40.0)
**Monobactams**	Aztreonam (ATM)	7 (46.7)	0 (0.0)	8 (53.3)
**Penicillins**	Ampicillin (AMP)	3 (20.0)	2 (13.3)	10 (66.7)
	Ticarcillin (TIC)	2 (13.3)	0 (0)	13 (86.7)
	Amoxicillin-Clavulanic Acid (AMC)	9 (60)	3 (20)	3 (20)
	Ampicillin-sulbactam (SAM)	9 (60)	6 (40)	0 (0)
	Piperacillin-tazobactam (TZP)	9 (60)	3 (20)	3 (20)
	Ticarcillin-Clavulanic Acid (TTC)	10 (66.7)	0 (0)	5 (33.3)
**Fluoroquinolones**	Ciprofloxacin (CIP)	7 (46.7)	2 (13.3)	6 (40.0)
	Levofloxacin (LEV)	9 (60.0)	2 (13.3)	4 (26.7)
**Cyclines**	Tigecycline (TGC)	13 (86.7)	0 (0.0)	2 (13.3)

High resistance rates were observed for penicillins used alone. Combinations with β-lactamase inhibitors, however, demonstrated improved activity. The susceptibility rates were 66.7% (10/15) for ticarcillin-clavulanate, 60% (9/15) for amoxicillin-clavulanate and piperacillin-tazobactam, and 60% (9/15) for ampicillin-sulbactam. Notably, 40% (6/15) of the isolates showed intermediate susceptibility to ampicillin-sulbactam.

Aminoglycosides showed good susceptibility against ETEC. Gentamicin demonstrated the highest susceptibility rate (86.7% (13/15)), followed by amikacin (73.3% (11/15)). Lower susceptibility was observed for kanamycin and tobramycin (both 53.3% (8/15)). Chloramphenicol and tigecycline each showed susceptibility rates of 86.7% (13/15).

Resistance patterns varied considerably across cephalosporin generations. Second-generation agents, particularly the cephamycin group, demonstrated the highest preserved activity, with cefoxitin exhibiting 100% susceptibility. In contrast, susceptibility to cefuroxime was reduced to 53.3% (*n* = 8). High resistance rates were observed for ceftazidime 60.0% (8/14) and cefotaxime 53.3% (8/15), while ceftriaxone showed a resistance rate of 40.0% (6/15). Furthermore, nearly half of the isolates (46.7% (7/15)) were resistant to the fourth-generation cephalosporin, cefepime (Table 4).

The susceptibility profile for the monobactam class was evaluated using Aztreonam. Similar to the patterns observed for fourth-generation cephalosporins, resistance to Aztreonam was high, reaching 46.7% (7/15). Only 46.7% (7/15) of the ETEC isolates remained fully susceptible, while 6.7% (1/15) exhibited intermediate susceptibility.

Fluoroquinolone resistance was frequent. Ciprofloxacin resistance was detected in 6 of 15 isolates (40.0% (6/15)), while resistance to levofloxacin was observed in 4 isolates (26.7% (4/15)).

In contrast to the older first-line agents, Chloramphenicol retained significant in vitro activity, with 86.7% (13/15) of isolates being susceptible. Similarly, Tigecycline, a cyclin often reserved for multidrug-resistant (MDR) Gram-negative infections, showed high efficacy with 86.7% (13/15) susceptibility. Resistance to tigecycline was low 13.3% (2/15), which is encouraging, though its clinical use in young children is generally restricted.

### Multidrug resistance and ESBL production

Based on standardized criteria, 11 of the 15 tested isolates (73.3% (11/15)) met the definition of multidrug resistance, exhibiting non-susceptibility to at least one agent in three or more antimicrobial classes. No extensively drug-resistant or pandrug-resistant profiles were identified.

Phenotypic screening suggested possible extended-spectrum beta-lactamase production in several isolates due to resistance to third-generation cephalosporins combined with preserved susceptibility to cefoxitin. Confirmatory testing using the double-disk synergy method identified one isolate as an ESBL producer.

**Fig. 1:**
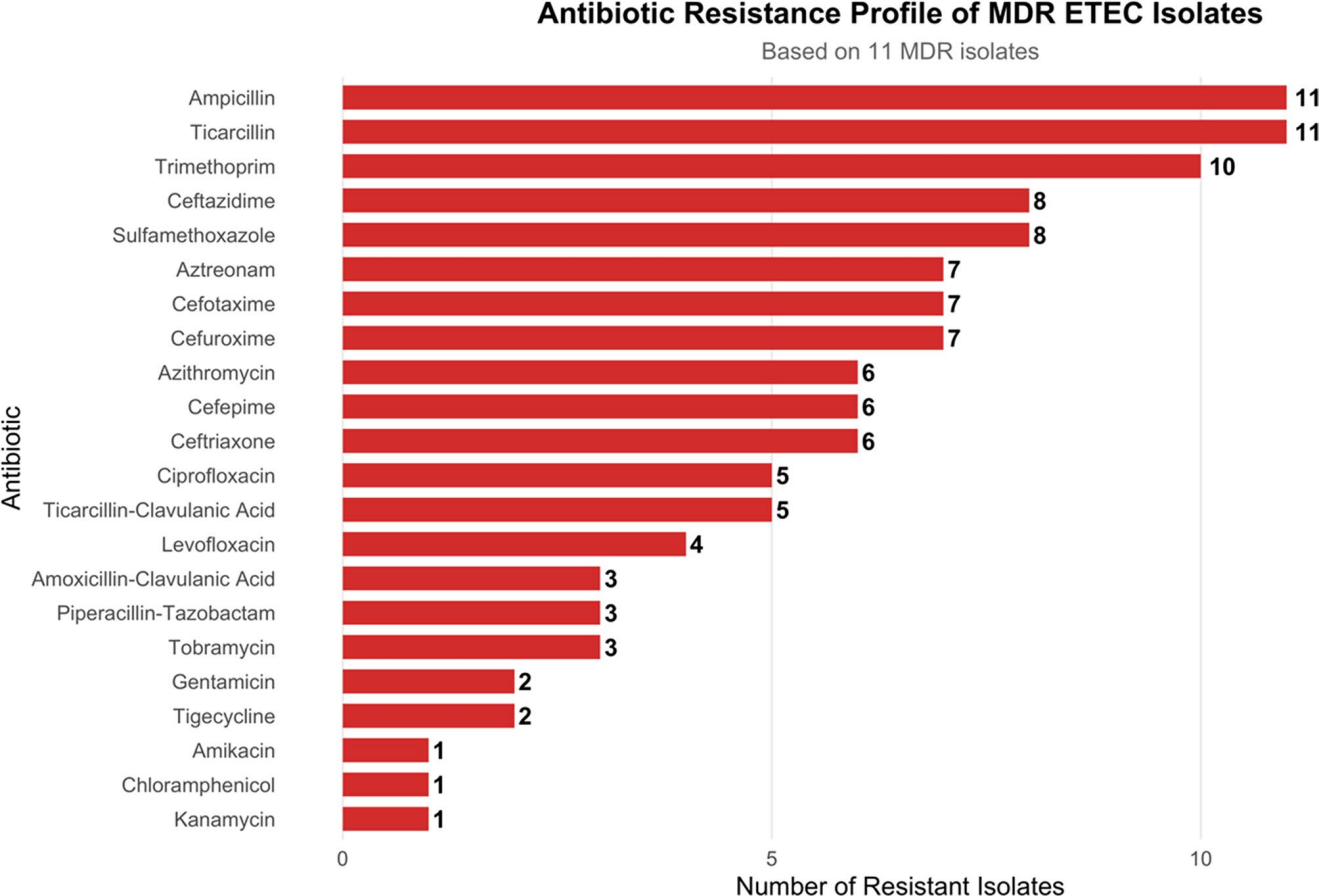
Distribution of resistant antibiotics among MDR isolates. Bar chart showing the number of isolates with resistance to each antibiotic, limited to MDR cases

Distinct resistance patterns observed among multidrug-resistant isolates are summarized in Table 5, and the frequency of resistance to individual antibiotics among MDR isolates is shown in Fig. 1.

**Table 5:** Resistance patterns among MDR ETEC isolates

Pattern	Resistant antibiotics	*n*	Frequency
**R1**	Amikacin, Ampicillin, Cefepime, Ceftriaxone, Cefuroxime, Piperacillin-Tazobactam, Ticarcillin	1	9.1%
**R2**	Amoxicillin-Clavulanic Acid, Ampicillin, Azithromycin, Aztreonam, Cefepime, Cefotaxime, Ceftazidime, Ceftriaxone, Cefuroxime, Ciprofloxacin, Kanamycin, Levofloxacin, Sulfamethoxazole, Ticarcillin, Ticarcillin-Clavulanic Acid, Tobramycin, Trimethoprim	1	9.1%
**R3**	Amoxicillin-Clavulanic Acid, Ampicillin, Azithromycin, Aztreonam, Cefepime, Sulfamethoxazole, Ticarcillin, Ticarcillin-Clavulanic Acid, Trimethoprim	1	9.1%
**R4**	Amoxicillin-Clavulanic Acid, Ampicillin, Azithromycin, Cefepime, Cefotaxime, Ceftazidime, Ceftriaxone, Cefuroxime, Ciprofloxacin, Piperacillin-Tazobactam, Sulfamethoxazole, Ticarcillin, Ticarcillin-Clavulanic Acid, Trimethoprim	1	9.1%
**R5**	Ampicillin, Azithromycin, Aztreonam, Cefotaxime, Ceftazidime, Chloramphenicol, Ciprofloxacin, Levofloxacin, Piperacillin-Tazobactam, Sulfamethoxazole, Ticarcillin, Tobramycin, Trimethoprim	1	9.1%
**R6**	Ampicillin, Azithromycin, Cefotaxime, Ceftriaxone, Cefuroxime, Ciprofloxacin, Gentamicin, Levofloxacin, Ticarcillin, Trimethoprim	1	9.1%
**R7**	Ampicillin, Azithromycin, Ceftazidime, Levofloxacin, Sulfamethoxazole, Ticarcillin, Tigecycline, Trimethoprim	1	9.1%
**R8**	Ampicillin, Aztreonam, Cefepime, Cefotaxime, Ceftazidime, Ceftriaxone, Cefuroxime, Gentamicin, Sulfamethoxazole, Ticarcillin, Tigecycline, Trimethoprim	1	9.1%
**R9**	Ampicillin, Aztreonam, Cefepime, Cefotaxime, Ceftazidime, Ceftriaxone, Cefuroxime, Ticarcillin, Ticarcillin-Clavulanic Acid, Trimethoprim	1	9.1%
**R10**	Ampicillin, Aztreonam, Cefotaxime, Ceftazidime, Ciprofloxacin, Sulfamethoxazole, Ticarcillin, Tobramycin, Trimethoprim	1	9.1%
**R11**	Ampicillin, Aztreonam, Ceftazidime, Cefuroxime, Sulfamethoxazole, Ticarcillin, Ticarcillin-Clavulanic Acid, Trimethoprim	1	9.1%

Note: Each pattern represents a unique combination of resistant antibiotics observed among MDR isolates

## Discussion

This hospital-based sentinel surveillance study provides updated data on the prevalence, toxin profiles, and antimicrobial resistance (AMR) patterns of enterotoxigenic Escherichia coli (ETEC) among children with acute diarrhoea in Ouagadougou. The study yields three principal findings: (1) an ETEC prevalence of 4.2%, confirming its persistent endemicity; (2) substantial genetic diversity, predominantly featuring heat-labile (LT, 50%) and heat-stable (STp, 43.75%) strains; and (3) high AMR rates, including 73.3% multidrug resistance (MDR) and 80% prevalence of isolates with phenotypic patterns suggestive of ESBL production. While these findings offer critical insights, it is important to note that the small number of confirmed ETEC isolates (*n* = 16, with 15 available for AST) warrants cautious interpretation of both the resistance estimates and toxin distribution patterns reported here.

The 4.2% prevalence aligns closely with prior local surveillance (3.2%–4.2%), underscoring relatively stable transmission dynamics over the past two decades [11, 23, 28]. Notably, culture-based detection likely underestimates the true burden compared to molecular methods like RLDT, which have shown ETEC positivity of 25% [11]. Infections were heavily concentrated among children aged 12–23 months (43.8%), consistent with age-specific distributions reported in the Global Enteric Multicenter Study (GEMS), where diarrhoeal pathogens including ETEC were frequently identified in this age group [29]. This vulnerability coincides with the transition from exclusive breastfeeding and increased environmental exposure, marking this age group as the primary target for future interventions.

The observed toxin profile potentially reveals important epidemiological patterns. While previous local studies frequently identified ST-dominant strains [11, 23], the near-balanced distribution between LT and STp in our cohort is consistent with broader West African pattern of genetic heterogeneity. Recent findings from the VIDA study link ST-producing strains to surface water consumption, whereas LT strains are associated with sanitation deficits [30]. This may suggest that the high STp burden in this study population is associated with water insecurity. Furthermore, the high proportion of STp-only isolates suggests that candidate ETEC vaccines should ideally incorporate both LT and ST antigens in order to achieve broader protective coverage.

Most concerningly, the documented AMR patterns suggest that several historically essential treatments may now be obsolete. Resistance to first-line agents, including trimethoprim (93.3%), sulfamethoxazole (86.7%), and amoxicillin (73.3%), likely precludes their empirical use. High resistance to third-generation cephalosporins (40%–57.1%) and the 80% prevalence of ESBL-suggestive phenotypes are consistent with the widespread regional dissemination of plasmid-mediated resistance determinants (e.g., CTX-M-type enzymes). Resistance to second-line options, including fluoroquinolones (up to 40%) and azithromycin (40%), further limits therapeutic flexibility. The 73.3% MDR rate observed in this study is higher than levels previously reported in Burkina Faso, where diarrheagenic *E. coli* studies documented substantial resistance and ESBL production; this increase may reflect several interconnected drivers, including over-the-counter antibiotic access, self-medication, incomplete treatment courses, and inappropriate antimicrobial use in both humans and animals [31–33].

Despite this, near-universal susceptibility was preserved for carbapenems (100%), amikacin (100%), and gentamicin (86.7%). Because these agents require parenteral administration and are typically reserved for severe, hospitalized cases, our findings suggest they must be rigorously protected through antimicrobial stewardship rather than used for outpatient management.

The adoption of the updated EUCAST definitions, particularly the reclassification of the “Intermediate” category to “Susceptible, increased exposure” (I), has important clinical and epidemiological implications for our findings [34]. Historically, results categorized as “I” were frequently interpreted by prescribers as indicating limited therapeutic value, which often led to these isolates being considered similar to resistant strains. Within the updated framework, it is now clearly established that antibiotics for which enterotoxigenic *Escherichia coli* isolates demonstrated “I” profiles may still represent effective therapeutic options when the dosing regimen is optimized to achieve adequate antimicrobial exposure at the site of infection.

This conceptual evolution is particularly relevant in our setting because it may help preserve and broaden the range of antimicrobials available for the management of pediatric patients. In addition, the integration of the Area of Technical Uncertainty (ATU) during antimicrobial susceptibility testing interpretation strengthens the reliability of our surveillance data by reducing the risk of misclassifying isolates with borderline inhibition zone diameters. Consequently, this approach ensures a more accurate estimation of the true burden of antimicrobial resistance.

Several limitations must be acknowledged. As noted previously, the small number of confirmed ETEC isolates (*n* = 16, with 15 tested for AMR) limits the precision of our resistance estimates. As a single-center urban study, results may not fully generalize to rural settings. Furthermore, due to the restricted sample size, we did not perform molecular characterization of specific ETEC AMR genes (e.g., *blaCTX-M*) and plasmid profiling; comprehensive genomic mapping is planned for a future, large-scale investigation. Finally, the lack of clinical outcome data and systemic antimicrobial exposure history prevented us from directly linking resistance profiles to treatment failures or specific patient risk factors.

Despite these limitations, this study provides critical baseline data using standardized EUCAST methodology. The findings demand urgent action across clinical, public health, and research domains. At the clinical and policy levels, national treatment guidelines, which have remained unchanged since 2015, urgently require revision based on contemporary local data. Robust diagnostic and antimicrobial stewardship must be prioritized to curb empirical broad-spectrum prescribing. From a public health perspective, comprehensive One Health frameworks and investments in water, sanitation, and hygiene (WASH) infrastructure are essential to disrupt transmission pathways.

Finally, regarding future research, subsequent investigations based on larger and more representative isolate collections should prioritize multicentre whole-genome sequencing of ETEC isolates to comprehensively map AMR mechanisms and track horizontal gene transfer events. Furthermore, the rigorous evaluation of multivalent ETEC vaccine candidates in West African paediatric populations remains a critical necessity.

## Conclusion

This sentinel surveillance study demonstrates that multidrug-resistant enterotoxigenic *Escherichia coli* represents a serious and rapidly evolving threat to the effective management of childhood diarrhoea in Ouagadougou, Burkina Faso. With 73.3% of ETEC isolates resistant to three or more antimicrobial classes, extremely high resistance rates to trimethoprim-sulfamethoxazole, ampicillin, and amoxicillin, and 80% of isolates exhibiting phenotypic profiles suggestive of ESBL production (with one isolate phenotypically confirmed), the therapeutic landscape for this common paediatric infection has substantially deteriorated. It is important to clarify that these findings are intended to inform regional surveillance and antimicrobial stewardship efforts rather than to serve as direct first-line treatment recommendations. Furthermore, the observed diversity of heat-labile and heat-stable toxin-producing strains has important implications for vaccine development, underscoring the need for broad-coverage multivalent vaccine candidates. Addressing this public health crisis requires urgent, coordinated action, including the revision of empirical treatment guidelines based on local surveillance data, strengthened antimicrobial stewardship, strict regulatory enforcement, investment in laboratory capacity, and intensified water, sanitation, and hygiene (WASH) interventions.

## Supplementary Information

Below is the link to the electronic supplementary material.

Supplementary Material 1

## Data Availability

The datasets used and/or analysed during the current study are available from the corresponding author on reasonable request.

## Abbreviations

AMR: Antimicrobial resistance
AST: Antimicrobial susceptibility testing
ATCC: American type culture collection
CAZ: Ceftazidime
ETEC: Enterotoxigenic *Escherichia coli*
ESBL: Extended-spectrum beta-lactamase
LT: Heat-labile toxin
ST: Heat-stable toxin
MDR: Multidrug-Resistant
EUCAST: European committee on antimicrobial susceptibility testing
