# Supplementary material for: Antimicrobial resistance and toxin profiles of enterotoxigenic Escherichia coli in children under five years old with diarrhoea in Ouagadougou, Burkina Faso: a sentinel surveillance study
Source: BMC Infect Dis. 2026 Jun 3;26:1249. doi: 10.1186/s12879-026-13722-8 (PMC13335261; doi:10.1186/s12879-026-13722-8)
Supplement: Supplementary file 1 — Supplementary Material 1 [file 12879_2026_13722_MOESM1_ESM.docx]

**Supplemental materials**

**1. Distribution of ETEC toxin profiles**

Among the 16 PCR-confirmed Enterotoxigenic *Escherichia coli* (ETEC) isolates, those producing heat-labile toxin (LT) alone were the most frequent (50.0% (8/16)). Strains producing heat-stable toxin and carrying the estA1 (STp) gene accounted for 7 isolates (43.8% (7/16)). A single isolate (6.2% (1/16)) was found to harbor both *eltB* (LT) and *estA2* (STh) genes. The detailed distribution by age group and sex is presented in Table 3 of the main manuscript.


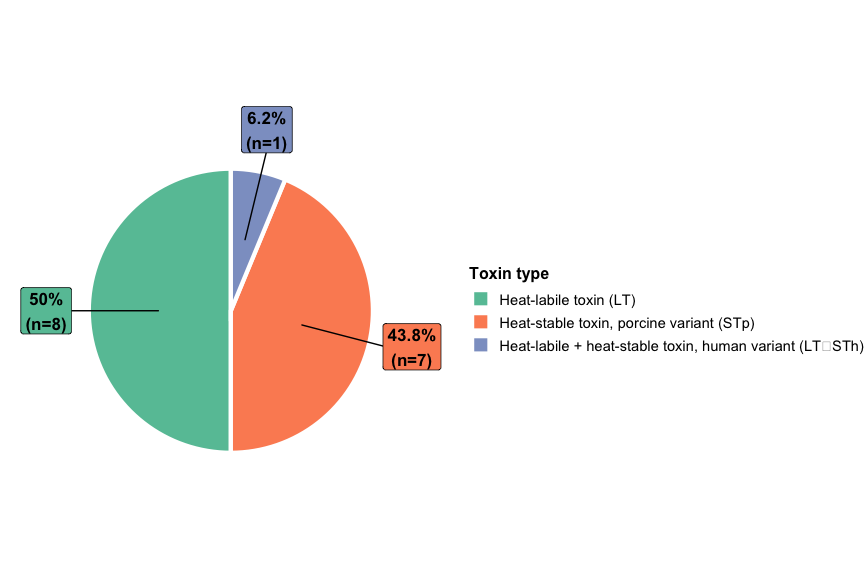


**Figure S1**. Distribution of ETEC toxin (n=16) among PCR confirmed ETEC from children under five years of age in Ouagadougou, Burkina Faso

**2. Antimicrobial susceptibility patterns**

**Penicillins and β-lactamase** **inhibitor combinations**

Isolates showed high resistance rates when penicillins were used alone. However, combinations with beta-lactamase inhibitors significantly improved antimicrobial activity. The susceptibility rates were 66.7% (10/15) for ticarcillin-clavulanate, 60.0% (9/15) for amoxicillin-clavulanate and piperacillin-tazobactam, and 60.0% (9/15) for ampicillin-sulbactam. Notably, 40.0% (6/15) of the isolates showed intermediate susceptibility to ampicillin-sulbactam.


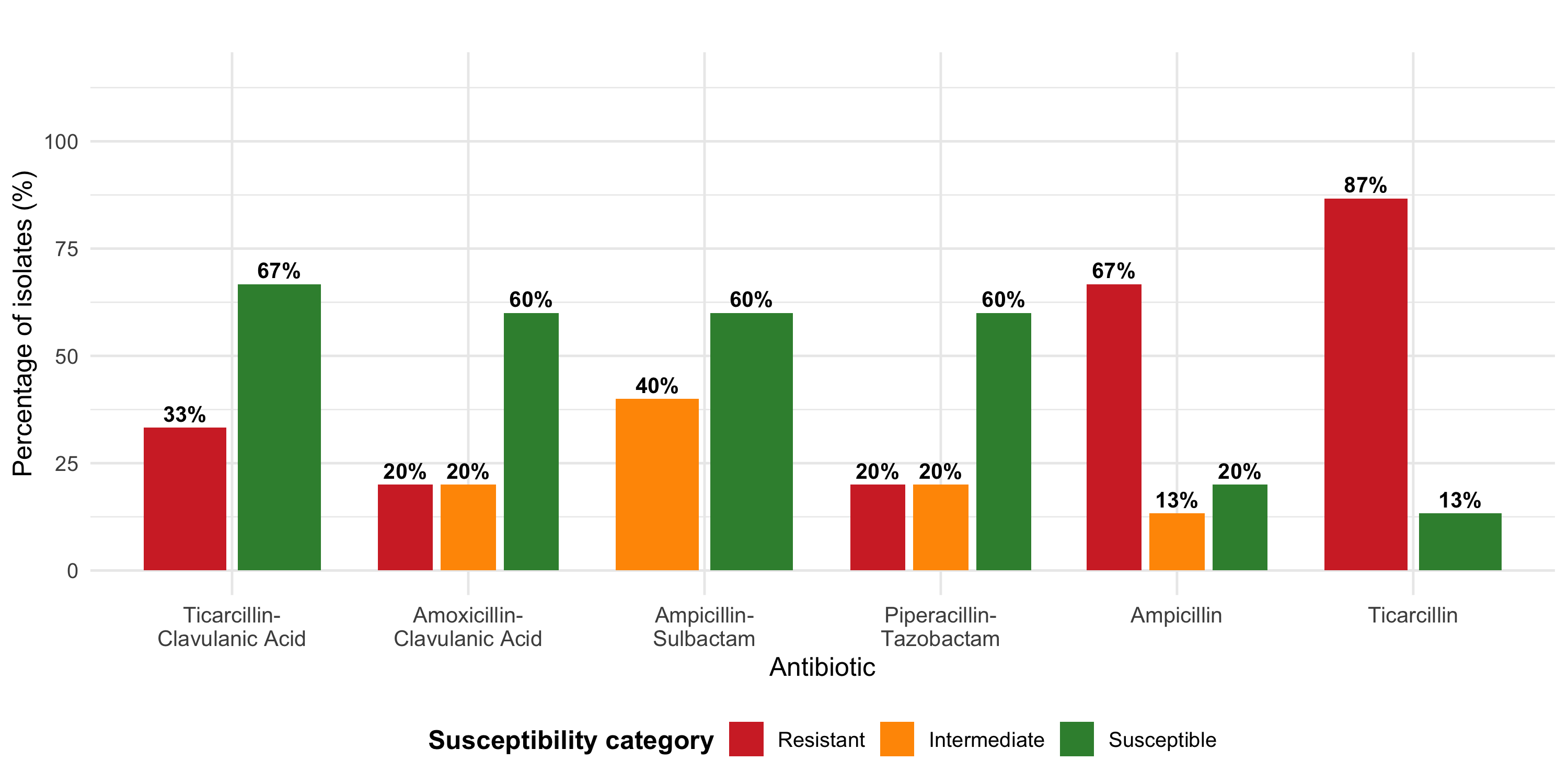


**Figure S2**. Resistance profile to penicillins and β-lactam/β-lactamase inhibitor combinations

Aminoglycosides showed good susceptibility against ETEC. Gentamicin demonstrated the highest susceptibility rate 86.7% (13/15), followed by amikacin 73.3% (11/15). Lower susceptibility was observed for kanamycin and tobramycin, each at 53.3% (8/15). Chloramphenicol and tigecycline each showed susceptibility rates of 86.7% (13/15).


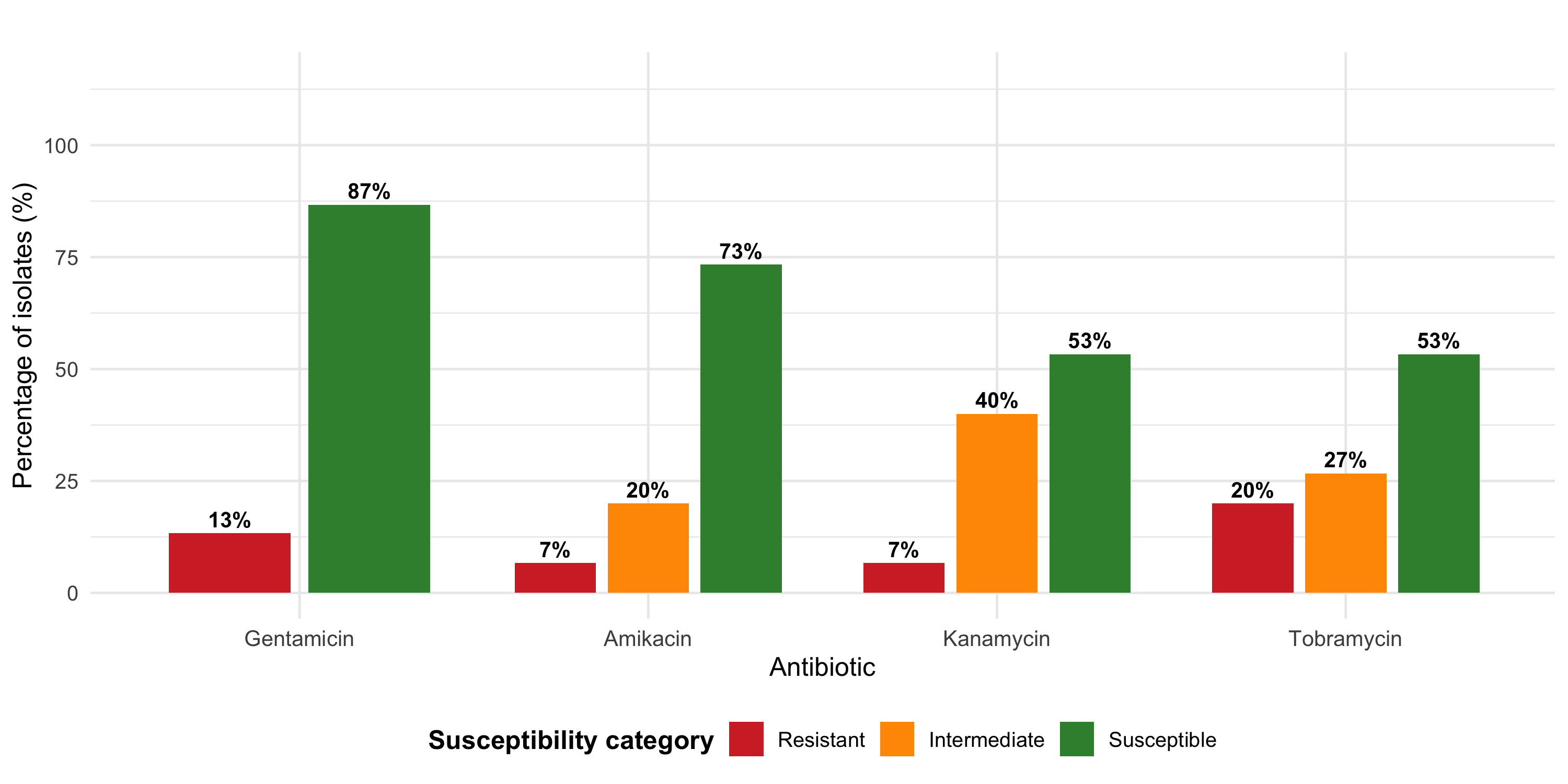


**Figure S3**. Resistance profile to aminoglycosides

Resistance patterns varied considerably across cephalosporin generations. Second-generation agents, particularly the cephamycin group, demonstrated the highest preserved activity, with cefoxitin exhibiting 100% susceptibility (15/15). In contrast, susceptibility to cefuroxime was reduced to 53.3% (8/15). High resistance rates were observed for ceftazidime 57.1% (8/14) and cefotaxime at 53.3% (8/15), while ceftriaxone showed a resistance rate of 40.0% (6/15). Furthermore, resistance to the fourth-generation cephalosporin cefepime reached 46.7% (7/15).

The susceptibility profile of the monobactam class was evaluated using aztreonam. Similar to the pattern observed for fourth-generation cephalosporins, resistance to aztreonam was high at 46.7% (7/15). Only 46.7% (7/15) of ETEC isolates remained fully susceptible, while 6.7% (1/15) exhibited intermediate susceptibility.

**
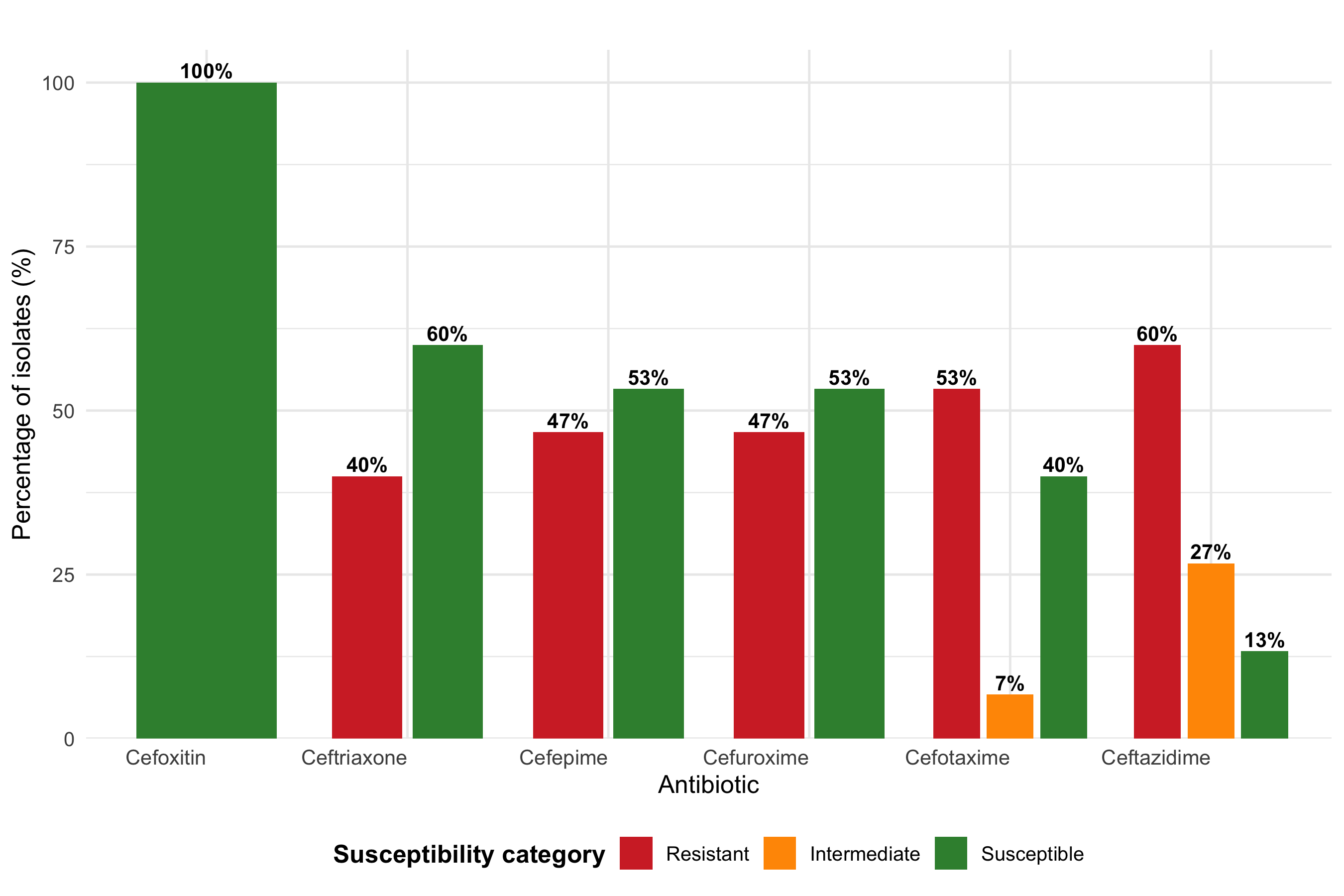
**

**Figure S4.** Resistance profile to cephalosporins
